# The influence of a pre-exercise sports drink (PRX) on factors related to maximal aerobic performance

**DOI:** 10.1186/1550-2783-7-12

**Published:** 2010-03-11

**Authors:** Allyn Byars, Susan Keith, Warren Simpson, Andy Mooneyhan, Mike Greenwood

**Affiliations:** 1 Department of Kinesiology, Angelo State University, San Angelo, TX 76909, USA; 2 Department of Exercise Science, Arkansas State University, State University, AR 72467, USA; 3 Department of Health, Human Performance and Recreation, Baylor University, Waco, TX 76798, USA

## Abstract

**Background:**

Pre-exercise sports drinks (PRX) are commonly used as ergogenic aids in athletic competitions requiring aerobic power. However, in most cases, claims regarding their effectiveness have not been substantiated. In addition, the ingredients in PRX products must be deemed acceptable by the athletic governing bodies that regulate their use in training and competition. The purpose of this study was to examine the effects of a modified PRX formulation (known as EM·PACT™) from earlier investigations on factors related to maximal aerobic performance during a graded exercise test. The modification consisted of removing creatine to meet the compliance standards set forth by various athletic organizations that regulate the use of nutritional supplements.

**Methods:**

Twenty-nine male and female college students varying in levels of aerobic fitness participated in a randomized crossover administration of PRX (containing 14 g/serving of fructose, medium-chain triglycerides, and amino acids mixed with 8 oz. of water) and placebo (PL) 30 minutes prior to performing a treadmill test with approximately one week separation between the trials. VO_2_max, maximal heart rate (HR), time to exhaustion (Time), and percentage estimated non-protein fat substrate utilization (FA) during two *a priori *submaximal stages of a graded exercise testing were evaluated.

**Results:**

The VO_2_max mean value of the PRX trial was significantly greater than the PL trial (P < 0.01). The mean value for Time was also observed to be greater for the PRX trial compared to PL (P < 0.05). Additionally, percentage of FA during submaximal stages of the exercise test was greater for PRX trial in comparison to PL (P < 0.01).

**Conclusions:**

The modified PRX formulation utilized in this investigation supports the findings of the previous investigation and its efficacy for enhancing indices of aerobic performance (specifically VO_2_max, Time, & FA) during graded exercise testing.

## Background

VO_2_max or the ability of the human body to use or consume oxygen for aerobic metabolism during exercise is an important predictor of athletic performance in endurance activities [1]. In addition, ventilatory threshold and the onset of blood lactate are considered to be even better indicators of an endurance athlete's capacity when examining the metabolic demands of middle distance runners and other similar athletes for aerobic power [2]. As such, the ability of an individual to reduce or tolerate more lactate production or the metabolic end product caused by the excessive metabolism of carbohydrates (CHO) is an important factor in the performance of endurance athletes as well as other sports that rely heavily upon aerobic metabolic pathways. Therefore, it is generally accepted that by using less CHO and more fat during activity with a concomitant decrease in lactate, aerobic performance of the individual should therefore be enhanced [3].

Previously, research has demonstrated that CHO ingestion during aerobic exercise can improve performance during exercise sessions lasting longer that 90 minutes performed at intensities greater than 70% VO2 max by preventing a decline in blood glucose concentration and facilitating glucose oxidation late, whereas the timing and type of CHO ingestion following exercise influences muscle glycogen restoration [4-6]. This information is especially important for endurance athletes since CHO type and blood glucose response is important in order to optimize CHO intake either pre or post exercise.

For example, CHO ingestion immediately prior to exercise has been reported to have a negative effect on exercise performance [7]. If an athlete consumes carbohydrate-rich foods or sport drinks within 60 minutes of the beginning of an endurance exercise performance, the glucose from the ingested food or drink enters the circulation within minutes of ingestion. The subsequent rise in blood glucose concentration causes the release of the hormone insulin, which assists in clearing glucose from the circulation. A peak in insulin concentration in the blood occurs at the time exercise begins. Consequently glucose uptake by the muscles reaches an abnormally high rate during the exercise performance. Therefore, the consumption of simple CHO, which are digested and absorbed quickly, can be detrimental to exercise performance [7].

This high clearance rate of glucose from the blood can potentially cause hypoglycemia which in turn can produce symptoms of acute fatigue. In summary, consuming high-glycemic CHO immediately before exercising causes blood glucose to rise rapidly (glycemic response) which may trigger excessive insulin release (insulinemic response) [8-10]. Endurance performance could be compromised by this rebound hypoglycemia, depressed fat catabolism, and possible earlier than expected depletion of glycogen stores.

In contrast, consuming low-glycemic CHO rich foods (starch with high amylose content or moderate glycemic CHO with high dietary fiber content) in the immediate 45-60 minute pre-exercise period allows for slower glucose absorption, reducing the potential for rebound glycemic response. Typically, the optimal forms of CHO have been combinations of glucose, fructose, sucrose, and maltodextrins with or without protein or amino acids and it has been further suggested that the glycemic index of food may be a key determining factor for when food is ingested relative to exercise participation [11-18].

Gastric emptying also affects fluid hydration and absorption of nutrients. Gastric emptying slows when ingested fluids contain a high concentration of particle in solution (osmolality) or possess high caloric content. The rate the stomach empties greatly affects intestinal absorption of fluid and nutrients. Little negative effect of exercise on gastric emptying occurs up to an intensity of about 75% of maximum, after which emptying rate slows [19]. Gastric volume, however, greatly influences gastric emptying; the emptying rate increases exponentially as fluid volume in the stomach increases. A major factor to speed gastric emptying (and compensate for any inhibitory effects of the beverage's carbohydrate content) involves keeping a relatively high fluid volume in the stomach. Consuming 150-250 ml of fluid immediately before exercise optimizes the beneficial effect of increased stomach volume on fluid and nutrient passage into the intestine. Prior research has also indicated that colder fluid emptied from the stomach at a faster rate than fluid at room temperature [3]. As a general rule, a 5 to 8% CHO-electrolyte beverage consumed during exercise in the heat contributes to temperature regulation and fluid balance as effectively as plain water by providing an intestinal energy delivery rate of approximately 5.0 kilocalories per minute in helping maintain glucose metabolism and glycogen reserves in prolonged exercise [20,21].

Another factor influencing absorption is the consumption of triglycerides composed of predominantly long-chain fatty acids (12-18 carbons) significantly delays gastric emptying. This affects the rapidity of fat availability negatively and also slows fluid and CHO replenishment, both crucial factors in high intensity endurance exercise. Consequently, the relatively slow rate of gastric emptying and subsequent digestion, absorption, and assimilation of long-chain triglycerides makes this energy source an undesirable supplement to augment energy metabolism [22].

Medium-chain triglycerides (MCTs) on the other hand provide a more rapid source of fatty acid fuel. MCTs are processed oils frequently produced for patients with intestinal malabsorption and tissue wasting diseases. Unlike long chain triglycerides, MCTs contain saturated fatty acids with 8-10 carbon atoms along the fatty acid chain. During digestion, lipase in the mouth, stomach, and intestinal duodenum hydrolyzes MCT to glycerol and medium chain fatty acids (MCFAs). Their water solubility allows MCFAs to move rapidly across the intestinal mucosa directly into the blood stream (portal vein) without first being transported slowly as chylomicrons by the lymphatic system as long chain triglycerides require [3].

Currently there are many sport drinks that help the body replenish CHO levels during exercise including pre-exercise formulas whose purpose is to promote the sparing of CHO by facilitating fat substrate utilization during exercise. Athletes, in particular those participating in sports requiring aerobic power, commonly use pre-exercise drinks (PRX) and/or other ergogenic aids prior to training workouts and competition. Although this practice is commonplace among athletes, many of the effectiveness claims associated with these products appear to lack solid evidence substantiated by appropriately designed research trials. Additionally, there may be concerns over the purity and amounts of the listed ingredients in the drink formulations including their distribution to athletes in meeting compliance standards set forth by various athletic organizations that regulate the use of nutritional supplements.

EM·PACT™ (Mannatech, Inc., Coppell, TX) is an energy and endurance pre-exercise drink (PRX) purported to increase oxygen consumption and improve fat utilization during aerobic activity. In previous studies, ingestion of EM·PACT™ significantly enhanced indices of maximal aerobic performance when compared to a water placebo as well as fat substrate utilization when compared to another nationally marketed sports drink [23,24].

Therefore, the purpose of this study was to examine the effects of a modified PRX formulation (modified version of EM·PACT™) from earlier investigations on factors related to maximal aerobic performance during a graded exercise test. Specifically, VO_2_max, heart rate (HR), time to exhaustion (Time), and estimated non-protein fat substrate utilization (FA) during two *a priori *submaximal stages of a graded exercise testing were evaluated. The modification consisted of removing creatine monohydrate to meet the compliance standards set forth by various athletic organizations that regulate the use of nutritional supplements.

## Methods

### Study Sample

In this investigation, twenty male and nine female recreationally active college students (n = 29), ages 19-29 years (21.79 ± 2.73), volunteered as subjects. Subjects signed university-approved informed consent statements in compliance with the institution's research review board on the campus in which the study was conducted. Descriptive characteristics of subjects are presented in Table 1.

**Table 1:** Descriptive characteristics of subjects (Mean ± Standard Deviation)

	Years	Height	Weight	Body Mass Index
Male (n = 20)	25.15 ± 2.43	180.73 ± 7.73	84.26 ± 15.73	25.79 ± 4.42
Female (n = 9)	21.00 ± 1.73	166.29 ± 4.21	68.02 ± 12.78	24.75 ± 5.74
Total (n = 29)	21.79 ± 2.73	176.24 ± 9.58	79.23 ± 16.52	25.47 ± 4.79

### Investigational Products

The modified version of EM·PACT™ is a citrus flavored energy and endurance pre-exercise drink containing a proprietary blend of the following ingredients (Total 14 g/dose): aloe vera extract, calcium citrate, L-carnitine, choline bitartrate, citric acid, fructose, lecithin, lemon oil powder, magnesium aspartate, magnesium succinate, MCTs, potassium aspartate, potassium succinate, silicon dioxide, gum ghatti, arabinogalactan, and glucosamine hydrochloride.

### Study Design

Subjects involved in this study were asked to submit to "two" maximal oxygen consumption tests (VO_2_max) within a week of each other with at least 48 hours between trials. Subjects were required to perform each maximal effort exercise test on a motor-driven treadmill. In addition, expired lung gases were examined for the purpose of determining the amount of oxygen used during exercise for VO_2_max. Expired lung gases were collected by sampling air exhaled from the mouth into a mouthpiece connected to sampling hoses and gas analyzers (Physiodyne, New York). The exercise intensity began at a low level and was advanced every three minutes by increasing the speed and incline of the treadmill belt using Bruce protocol [25]. During the test, heart rate and time were measured continuously while blood pressure and ratings of perceived exertion (RPE) were measured toward the end of each three minute stage. VO_2_max was considered to have been achieved if the subject met at least two of the following criteria: 1) an RER equal to or greater than 1.15 2) plateau of the VO_2 _during the last stage of exercise 3) maximal heart rate within ± 10 beats per minutes of predicted values.

Prior to test participation, subjects were asked to adhere to the following pre-test instructions: 1) Wear comfortable, loose-fitting clothing 2) Drink plenty of fluids over the 24-hour period preceding the test 3) Avoid food, tobacco, alcohol, and caffeine for 3 hours prior to taking the test 4) Avoid exercise or strenuous physical activity the day of the test 5) Get an adequate amount of sleep (6 to 8 hours) the night before the test [25].

Each subject arrived thirty-five minutes prior to each exercise trial and was given either the recommended dosage (1 Tablespoon/14 g per 8 ounces/.24 L water) of PRX or a placebo (PL) [citrus flavored water] thirty minutes prior to test participation. Administration of PRX and PL trials were randomized with half of the participants ingesting the PL during the first trial and PRX during their second trial with the order reversed for the remaining subjects. Total participation time for each test was approximately 1 hour. The PRX supplement (EM·PACT™) was provided from Mannatech, Inc., Coppell, TX in sealed bottles and an open-label format with an unbiased assistant (not involved in the testing or data analysis) mixing the PRX and providing it to the subjects. Both the PRX and PL were provided by an assistant blinding both subjects and investigators as to the order in which the PL or PRX was ingested. At the end of the study investigators were provided information as to the order in which the subjects were provided either the PRX or PL.

Data were analyzed using a 2 × 2 (groups by trials) repeated measures ANOVA. VO_2_max (ml·kg^-1^·min^-1^), HR (beats per minute), Time (minutes), and FA (%) during two *a priori *submaximal stages of graded exercise testing were examined by gender as well as by the entire group of subjects. An alpha level of 0.05 was used in determining statistical significance. Statistical analyses were performed using SPSS for Windows version 16.0 statistical package (SPSS, Inc., Chicago, IL) [26]. Data are presented as means ± standard deviations (SD) for PL and PRX trials.

### Ethics Approval

Institutional Review Board approval was granted by the institution where the investigation was conducted (Angelo State University in San Angelo, TX) preceding the commencement of the study.

## Results

**Figure 1:**
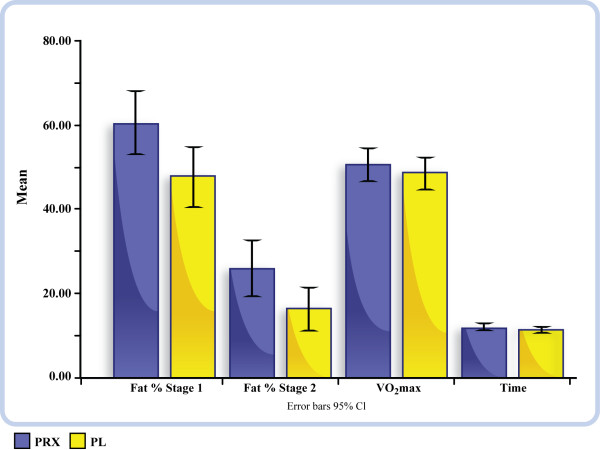
**results graph**.

Initial results indicated significant mean differences in VO_2_max (ml·kg-1·min-1) between PRX (50.49 ± 10.02) and PL (48.49 ± 9.91) trials for the total group (p = 0.007), which was not affected by gender (p > 0.05). Overall differences in the various parameters are depicted in Figure 1.

No significant mean differences in maximal HR (beats·min-1) were found between the PRX (188.66 ± 9.48) and PL (189.66 ± 9.49) trials for all subjects nor for either gender (p > 0.05). Significant mean differences in Time were found between the PRX (11.74 ± 1.72) and PL (11.44 ± 1.65) trials for all subjects (p = 0.034) and was not affected by gender (p > 0.05).

Significant mean differences in FA were found between PRX (60.30 ± 18) and PL (47.62 ± 17.08) in stage 1 (3rd minute, p = 0.009) and in stage 2 (6th minute, p = 0.008), PRX (25.79 ± 16.11) and PL (16.42 ± 112.37) of the graded exercise protocol for all subjects and was not affected by gender (p > 0.05). Overall differences in the two stages are depicted in FIGURE 1. Differences in mean values among all of the reported variables are displayed in Table 2.

**Table 2:** Mean and standard deviations of various parameters

	PL		PRX	
**Variable (n = 29)**	**Mean ± Standard Deviation**	**95% C.I**.	**Mean ± Standard Deviation**	**95% C.I**.

VO_2_max (ml^.^kg^-1.^min^-1^)	48.49 ± 9.91	44.72-52.26	50.49 ± 10.02**	46.68-54.30

Time (minutes)	11.44 ± 1.65	10.80-12.08	11.74 ± 1.72*	11.07-12.41

HR (beats^.^min^-1^)	188.66 ± 9.48	185.09-192.29	189.66 ± 9.49	186.04-193.27

FA (%) Stage 1	47.62 ± 17.08	40.57-54.68	60.30 ± 18.11**	52.83-67.78

FA (%) Stage 2	16.42 ± 12.37	11.31-21.53	25.79 ± 16.11**	19.14-32.44

## Discussion

The main findings of this study were that aerobic performance, specifically mean VO_2_max, FA, and Time were significantly (p < .05) improved by ingestion of PRX prior to graded exercise testing. In particular, overall increases were observed in VO_2_max (4.1%), non-protein FA (stage 1 = 26.6%, stage 2 = 57.1%), and time to exhaustion (2.6%). The findings of the present study support an earlier investigation of the PRX used in this study without the inclusion of creatine monohydrate in the drink formulation [23]. In addition, non-protein FA was also similar to an earlier study involving the PRX used in this investigation compared to another nationally marketed sports drink during the early stages of maximal exercise treadmill protocol [24]. Although the differences in the aforementioned parameters (VO_2_max & Time) between PRX and PL trials were not as marked as the original investigation, the inclusion of subjects with higher levels of fitness in the later study may account for this disparity since the window of potential improvement in these individuals may not have been as great [23]. The results of this study also support the use of the PRX as examined in this investigation in tests of aerobic power. This appears to be consistent with earlier reports of ingesting a PRX consisting of low glycemic sugars before exercise including a recent study examining the effects of CHO on performance changes (i.e., time and fuel substrate utilization) and overreaching in trained cyclists [12-18,27].

Improvement in time to exhaustion claims may also be substantiated as the data of this investigation support another investigation in which a mixture of CHO and medium-chain triglycerides (MCTs) resulted in increased aerobic function as marked by increases in length of time trials to exhaustion [6,28]. It is also fairly common for the nutritional supplement industry to market MCTs as fat burners, energy sources, glycogen sparers, and muscle builders to fitness and sports enthusiasts. Although MCTs do not inhibit gastric emptying as does common fat, conflicting research supports the efficacy of using MCTs solely or in combination with CHO as a means of improving oxidation during exercise and because of its limited amount in the formula studied in this investigation, its contribution may be minimal [29,30]. However, Subsequent research investigating possible metabolic and ergogenic effects of combining MCTs and CHO may have value. For instance, researchers in a recent study examining the effects of ingesting small additional amounts of MCTs in the diet for two weeks found that recreational athletes increased their time to exhaustion at pre-determined workloads along with increases in fat oxidation while yet another investigation reported no further improvements when combined with CHO [31,32]. As such, additional research may be needed in regards to the concentrations and timing of MCTs and CHO in the diet/supplements and their role in human performance.

## Conclusions

As a result of these findings, it was concluded that aerobic performance, specifically VO_2_max, Time, and FA may be significantly improved by ingestion of PRX 30 minutes prior to exercise testing. The modified PRX formulation utilized in this investigation supports the previous investigation and its efficacy for enhancing indices of aerobic performance (VO_2_max, Time, and FA) during graded exercise testing, indicating that creatine monohydrate is not necessary for product effects. During aerobic exercise bouts, the combined results of this investigation may provide meaningful practical applications for coaches and athletes alike regarding ergogenic hydration options. Future research is warranted investigating the efficacy of PRX with further emphasis on other variables such as fuel substrate utilization, gender differences, fitness levels, comparisons with other products, as well as use under various environmental and competitive conditions including timing of ingestion (both long and short-term), and the intensity/duration of various activities. Although the results of this investigation favor using this particular PRX, caution should be taken regarding the findings as further research is needed to provide a feasible scientific rationale why any significant finding occurred based on the content of the product. To the author's knowledge, no previous investigations have shown similar significant acute findings utilizing a proprietary blend of ingredients primarily designed for use as a concentrated sports drink.

## Competing interests

The authors declare that they have no competing interests.

## Authors' contributions

AB developed the concept of the study, contributed to its design, data collection, statistical analysis, and manuscript preparation. SK & WK contributed in the design of the study, data collection, and manuscript preparation. AM & MG provided background work for the manuscript and contributed to its preparation.

All authors have read and approved the final manuscript.
